# Correlation Between Fine Needle Aspiration Cytology (FNAC) and Permanent Histopathology Results in Salivary Gland Masses

**DOI:** 10.7759/cureus.13976

**Published:** 2021-03-18

**Authors:** Ghassan Z AlGhamdi, Ali K Alzahrani, Hisham Saati, Hussam M Algarni, Khalid A Alshehri, Muhannad Baroom, Baraa I Awad, Mohammed Algarni, Hadi Afandi Al-Hakami

**Affiliations:** 1 Medicine, College of Medicine, King Saud Bin Abdulaziz University for Health Sciences, Jeddah, SAU; 2 Otolaryngology - Head and Neck Surgery, King Saud Bin Abdulaziz University for Health Sciences, Jeddah, SAU; 3 Orthopaedics, King Saud Bin Abdulaziz University for Health Sciences, Jeddah, SAU; 4 Otolaryngology - Head and Neck Surgery, College of Medicine, King Saud Bin Abdulaziz University for Health Sciences, Jeddah, SAU; 5 College of Medicine, Department of Otolaryngology - Head & Neck Surgery, King Saud bin Abdulaziz University for Health Sciences, King Abdullah International Medical Research Center, Ministry of National Guard Health Affairs, Jeddah, SAU

**Keywords:** salivary gland mass, fine needle aspiration, cytology, cytopathology

## Abstract

Objective

Fine-needle aspiration cytology (FNAC) has been widely accepted as a diagnostic safe method for preoperative assessment of salivary gland lesions. This diagnostic tool is inexpensive, easy to perform, relatively painless and it provides useful information to differentiate between benign and malignant salivary gland tumors that helps in the management and surgical planning. This study was undertaken to compare FNAC results with permanent histopathological findings of salivary gland tumors in order to assess its diagnostic accuracy.

Materials and methods

A total of 37 archived salivary gland FNAC specimens collected between January 2001 and January 2018 were correlated with proven histopathology findings. Sensitivity, specificity, positive and negative predictive values, and diagnostic accuracy were calculated. False negative and false positive cases were determined.

Results

There were 20 female and 17 male patients. Parotid tumors count for 62.2% and submandibular tumors 37.8%. All cases of malignancy on FNAC were proven to be malignant on the final pathology findings. All cases that were suspicious for malignancy on FNAC were proven to be malignant as well. In addition, three false negative cases were seen and no false positive cases among all FNAC cases. In our series, the overall sensitivity and specificity were 90.3% and 100%, respectively. The positive and negative predictive values were 100% and 57.1%, respectively. The diagnostic accuracy was 91.4%.

Conclusion

This study demonstrated that FNA cytology of the salivary gland is a useful technique for diagnosis of salivary gland lesions. Insufficient cellularity was the most important factor that resulted in incorrect cytological interpretation.

## Introduction

Salivary gland tumors include a variety of different morphological and pathological groups of neoplasms that are not common and the associated histopathology is extremely varied and complex. These varieties of salivary gland lesions include epithelial neoplasms, non-epithelial lesions, lymphomas, metastatic tumors and non-neoplastic lesions. This variation further contributes to the diagnostic difficulty [1]. In the United States, new cases are estimated to be nearly 2.5‒3.0 cases per 100,000 per year [2]. Malignant salivary gland neoplasms account approximately 3‒5% of all head and neck tumors and more than 0.5% of all malignancies [2]. Fine-needle aspiration cytology (FNAC) is used widely in the preoperative assessment of head and neck tumors including salivary gland tumors. This diagnostic procedure is a safe, quick, simple, inexpensive, well-accepted and well-tolerated by patients. Although head and neck lesions are more easily accessible by FNA, diagnosis of salivary gland lesions by FNA is a controversial issue [3-5]. This method can help to differentiate between benign and malignant salivary gland tumors. It was found that FNAC in comparison to histological findings of parotid gland lesions in particular had a concordance rate of 86%, a specificity of 98%, a sensitivity of 86%, and a diagnostic accuracy of 94% [1]. Despite these findings, there are still concerns about its use as a main method of diagnosis due to its relatively low sensitivity in malignancy cases and its variation in results [6].

In addition to the insufficient cellularity, there are several confounding cytologic factors that make some FNA specimens difficult to interpret. Furthermore, some salivary gland malignancies can only be diagnosed by the presence of capsular invasion, which is not assessable by FNA [7]. Up to our knowledge, there is no study in Saudi Arabia addressing the correlation between fine needle aspiration cytology and histopathological results of salivary gland lesions. The aim of this retrospective study is to correlate FNAC results with the final histopathological findings of salivary gland tumors and to determine the diagnostic yield of FNA cytology and its accuracy.

## Materials and methods

Patients and specimens

This is a retrospective study consisting of 37 FNA specimens of salivary glands obtained from the archives of the cytology laboratory and the health information system "BESTCare", which were verified by histopathological diagnosis. Those patients had presented to ENT-Head and Neck clinics and had a parotidectomy or submandibular gland surgery at the King Abdul Aziz Medical City, Jeddah in a span of 17 years from January 2001 to January 2018. The study was approved by King Abdullah International Medical Research Center and IRB ethical approval was given with ID: SP20/123/J. Specimens with lack of pathologic confirmation or unsatisfactory cytology, such as blood only or no cells, were excluded. In addition, specimens not treated by surgery were also excluded. The FNA cytologic results were classified into four diagnostic categories: benign tumor, benign lesion, suspicion of malignancy, and malignant. Benign lesions refer to atypical cells that cannot be differentiated between reactive or neoplastic activity. The pathology of surgical specimens was classified into two main diagnostic categories: benign and malignant. For each individual patient we compared the histopathology of the permanent surgical specimens with the preoperative cytology of the FNAC samples.

Statistical analysis

For data analysis we used SPSS Base version 20.0. (IBM Corp., Armonk, NY). The sensitivity, specificity, positive predictive value (PPV), negative predictive value (NPV), and overall accuracy of FNAC were calculated using standard statistical methods to differentiate between benign and malignant disease. The qualitative variables presented as frequencies and percentages and the quantitative variables as mean and standard deviation. In order to compare the qualitative variables, we used the chi-square test. For comparing the quantitative variables, the t-test was used.

## Results

Among 37 specimens verified by pathology, there were 20 female and 17 male patients. The female-to-male ratio was approximately 1 to 1.2. The age range was 13 months to 93 years with a mean age of 56 years. Parotid tumors were 62.2% and submandibular tumors were 37.8%. Table 1 shows the general description of the salivary gland lesions. FNA cytologic diagnoses showed malignancy in 28 cases (75.7%), suspicion of malignancy in two (5.4%) and benign tumors and lesions in seven (18.9%). Table 2 shows the details of FNA cytology results in each category. The final pathologic diagnoses showed 33 malignant tumors (89.2%) and four (10.8%) benign tumors. Table 3 demonstrates the final histopathological results of each category. The most common benign tumor in both FNAC and final histopathology was pleomorphic adenoma. The most common malignancies in both were mucoepidermoid and adenoid cystic carcinoma. The correlation between FNAC and final pathologic results is demonstrated in Table 4. Three cases were false negative on FNAC, one case of pleomorphic adenoma and the other two were benign lesions. All cases of malignancy on FNAC were proven to be malignant on the final pathology. All cases that were suspicious for malignancy on FNAC were proven to be malignant as well. No false positive cases were seen among all FNAC cases. In our series, the overall sensitivity and specificity were 90.3% and 100%, respectively. The positive and negative predictive values were 100% and 57.1%, respectively. For determining the diagnostic accuracy of FNAC, the results of FNAC of each case were compared with the final histopathological diagnosis, as illustrated in Table 4. The diagnostic accuracy was 91.4%.

**Table 1:** General description of patients with salivary gland masses

Variables	No. (%)
Gender	
Male	17 (45.9)
Female	20 (54.1)
Median age	56 years
Site	
Parotid gland	23 (62.2)
Submandibular gland	14 (37.8)

**Table 2:** Categorization of FNAC results FNAC: Fine needle aspiration cytology

Category	No. (%)
Benign tumors	
Pleomorphic adenoma	5 (13.5)
Benign lesions	2 (5.4)
Suspicious for malignancy	2 (5.4)
Malignancy	28 (75.7)
Mucoepidermoid carcinoma	10 (27.0)
Adenoid cystic carcinoma	7 (18.9)
Acinic cell carcinoma	2 (5.4)
Squamous cell carcinoma	2 (5.4)
Undifferentiated carcinoma	2 (5.4)
Poorly differentiated carcinoma	1 (2.7)
Epithelial-myoepithelial carcinoma	1 (2.7)
Hemangiopericytoma	1 (2.7)
Neuroblastoma	1 (2.7)
Rhabdomyosarcoma	1 (2.7)

**Table 3:** Categorization of final histopathological specimens

Category	No. (%)
Benign tumors	
Pleomorphic adenoma	4 (10.8)
Malignancy	33 (89.2)
Mucoepidermoid carcinoma	11 (29.7)
Adenoid cystic carcinoma	9 (24.3)
Acinic cell carcinoma	3 (8.1)
Squamous cell carcinoma	2 (5.4)
Undifferentiated carcinoma	1 (2.7)
poorly differentiated carcinoma	2 (5.4)
Epithelial-myoepithelial carcinoma	1 (2.7)
Lymphoepithelial carcinoma	1 (2.7)
Hemangiopericytoma	1 (2.7)
Neuroblastoma	1 (2.7)
Rhabdomyosarcoma	1 (2.7)

**Table 4:** Correlation of FNA cytologic and final histopathologic diagnosis of 37 salivary gland lesions FNA: Fine needle aspiration

Total	Cytology				Histopathology
	Malignant tumors	Suspicious for malignancy	Benign lesions	Benign tumors	
4	0	0	0	4	Benign tumors
33	28	2	2	1	Malignant tumors

## Discussion

For the diagnosis of salivary gland neoplasms, fine needle aspiration is a safe and cost-effective method. FNAC can be done in an outpatient setting with low risk of complications [8]. The main goal of FNA is to determine if a mass is inflammatory and/or reactive, benign or malignant neoplasm and if possible, to render a specific diagnosis. The preoperative information concerning the tumor type can be informative to plan the best surgical approach. Close cooperation between the clinician and an experienced cytopathologist provides good outcome in FNA procedures. FNA in salivary gland lesions is one of the most difficult areas in cytopathology due to the overlapping morphologic patterns in many benign and malignant salivary gland neoplasms. Furthermore, histological patterns may show various differences within the same tumor [9,10]. Even though it has commonly become one of the initial tests to evaluate parotid masses, there is still some controversy regarding its effectiveness due to its low sensitivity in differentiating benign from malignant tumors [6].

In our study, we observed that most of the cases were malignant which could be explained by the referral system of our oncology center. Three cases were false negative on FNAC, one case of pleomorphic adenoma and other two were benign lesions. All cases of malignancy on FNAC were proven to be malignant on the final pathology. All cases that were suspicious for malignancy on FNAC were proven to be malignant as well. No false positive cases were seen among all FNAC cases. In our series, the overall sensitivity and specificity were 90.3% and 100%, respectively. The positive and negative predictive values were 100% and 57.1%, respectively. The diagnostic accuracy was 91.4%.

Our results are comparable with the other literature regarding the effectiveness of FNAC in the evaluation of salivary gland masses. A review of the literature showed an FNAC sensitivity ranging from 54% to 92% and a specificity of 86‒100% [8].

In one study where they reviewed 129 patients who underwent parotidectomy, similar results were shown with FNAC having a sensitivity of 84% and a specificity of 94%. The overall accuracy of FNAC in their study was around 94% [1]. Another study which was a retrospective chart review of 114 patients showed that FNAC had a sensitivity of 73% and a specificity of 97%. The overall accuracy of FNAC in that study was 95% [8]. Furthermore, the accuracy of FNAC in recent literature has ranged from 84% to 97% in comparison with our study which was 91.4% [11-13].

To answer our research question in regard to the accuracy of FNAC in correlation with histopathologic findings, it is fair to say that FNAC has high specificity that can help the surgeon in the counseling and management of the patients. Fine needle aspiration cytology of salivary gland lesions in particular needs expert cytopathologists and a collaborative team discussion between the pathologist, the surgeon and the radiologist to reach the precise diagnosis.

Some of the limitations of this study was the retrospective nature of it. The small number of specimens was another limitation. Therefore, we recommend further studies with larger samples to fully assess the accuracy of FNAC for diagnosing salivary gland masses in Saudi Arabia.

## Conclusions

FNAC is a reliable method with high specificity for malignant parotid gland tumors, and provides the surgeon with valuable information in preoperative diagnostics. FNAC is an easy to perform, inexpensive and cost-effective test with high overall accuracy in diagnosing benign and malignant salivary gland neoplasms. In our series, the overall sensitivity and specificity were 90.3% and 100%, respectively. Thus, this method provides several advantages for both the clinicians and the patients.
